# Impact of Intestinal Ultrasound on Classification and Management of Crohn's Disease Patients with Inconclusive Colonoscopy

**DOI:** 10.1155/2016/8745972

**Published:** 2016-04-19

**Authors:** Rune Wilkens, Kerri L. Novak, Eleonore Lebeuf-Taylor, Stephanie R. Wilson

**Affiliations:** ^1^Department of Radiology, University of Calgary, Calgary, AB, Canada T2N 2T9; ^2^Department of Medicine, Division of Gastroenterology, University of Calgary, Calgary, AB, Canada T2N 2T9; ^3^Diagnostic Centre, University Research Clinic for Innovative Patient Pathways, Silkeborg Regional Hospital, 8600 Silkeborg, Denmark; ^4^Department of Hepatology and Gastroenterology, Aarhus University Hospital, 8000 Aarhus C, Denmark

## Abstract

*Background and Aims*. We aim to evaluate the benefit of ultrasound in the assessment of Crohn's disease and to demonstrate its potential contribution to disease management.* Methods*. We conduct a retrospective review of adult patients with Crohn's disease examined with sonography and colonoscopy within 30 days. Study patients were identified in whom colonoscopy did not access a pathological segment, detected and evaluated by ultrasonography. Changes in management were predominantly attributed to ultrasound in those cases where the diseased segment was not assessed on endoscopy.* Results*. From 115 patients with temporally related ileocolonoscopy and ultrasound, 41 had disease fully assessed on ultrasound only, with complications in 26/41. Twenty-nine of 41 had mild or no endoscopic inflammation with moderate or severe disease on ultrasound at the same segment or at a segment proximal to the reach of the endoscope. Changes in management were significantly attributed to ultrasound in 22 of these 29 patients.* Conclusion*. The benefit of cross-sectional imaging is invaluable for the comprehensive assessment of bowel not shown on ileocolonoscopy. Ultrasound may make a significant contribution to correct classification of disease extent and severity of Crohn's disease. Prospective studies are needed to further understand the contribution of US in patient management.

## 1. Introduction

Crohn's disease (CD) is a chronic transmural inflammatory disease that may affect any portion of the gastrointestinal tract, with a predilection for the small intestine [1]. With time, the bowel wall becomes thick due to chronic inflammatory infiltrate, muscle cell proliferation, and fibrotic change [2, 3], often leading to stenosis and penetrating disease with a high requirement for surgical intervention [4].

Currently, ileocolonoscopy is considered the gold standard for diagnosing and monitoring CD. However, passage of the colonoscope can be hindered by a number of factors, such as narrowing of the intestinal lumen secondary to oedema or stricture, patient discomfort, and technical complexity of the procedure. Furthermore, the reach of the conventional examination is limited, at best, to the distal terminal ileum (TI). Consequently, diseased segments proximal to the reach of the endoscope are undetected. Failure to intubate the TI on ileocolonoscopy has been found to occur in 5–19% of patients with inflammatory bowel disease (IBD) [5, 6]. Ileocolonoscopy is also invasive and imparts risk of perforation.

Cross-sectional imaging is increasingly recognised as central to the evaluation of CD [7]. It is noninvasive and, therefore, an ideal component at diagnosis and routine monitoring of disease. Computed tomography enterography (CTE) and magnetic resonance enterography (MRE) are established modalities for this purpose [8].

Ultrasonography (US) with colour Doppler imaging (CDI) is a radiation-free, widely available, and relatively inexpensive cross-sectional imaging modality that can also provide direct, real-time information about the gastrointestinal tract for the evaluation of extent and severity of disease [9]. With a sensitivity reported to be consistently over 90% with adequate training [10] and a specificity of 91% for the detection of CD [11], US is an important tool in emergency settings [1], at time of diagnosis [7], and at follow-up [12]. Established sonographic findings suggestive of CD are gastrointestinal mural thickening exceeding 3 mm, increased hyperaemia detected on CDI, increased mesenteric fat, and the presence of large lymph nodes [9, 13–15]. US is a useful tool to assess complications such as stricturing and penetrating disease, the extent of which may not be seen on colonoscopy.

Two recent retrospective studies show the benefit of performing cross-sectional imaging of the intestine beyond the reach of normal ileocolonoscopy [5, 6]. However, ultrasound is not included in either study. Although bowel US is proven to be a strong diagnostic tool in patients with CD, there is limited literature describing how it may influence clinical decision-making for CD patients [16]. The purpose of this study, therefore, is to investigate the potential contribution of bowel US to the detection and correct classification of inflammation in CD and to examine a possible influence on disease management by way of evaluating patients in whom the full extent of disease is evident on US only.

## 2. Materials and Methods

### 2.1. Study Design and Patient Population

This retrospective study was approved by the Institutional Ethics Review Board. The necessity for informed consent was waived. From the Picture Archiving and Communication System (PACS) at our tertiary referral center, we reviewed the time interval between June 2007 and April 2011, identifying patients with established or suspected CD who underwent bowel US with CDI. Inclusion criteria were colonoscopy within 30 days of the US study. Exclusion criteria were age less than 17 years and no final confirmed diagnosis of CD.

Sonography and colonoscopy reports were reviewed in all patients. We identified those in whom a complete ileocolonoscopy was performed and those in whom a diseased segment of bowel, visualised on ultrasound, was not accessed on colonoscopy due to either location (proximal small bowel) or physical limitation such as stenosis. In the group of patients with failure to intubate the TI or anastomosis, all patients with US-reported inflammation proximal to that detected endoscopically were noted. All patients in whom the full extent of disease was visualised on US only were categorised as the ultrasound group. The remainder of the patients, in whom US and colonoscopy showed concordant results or in whom lesions were described on colonoscopy only, were categorised as the endoscopy group.

Demographic information, US and endoscopic features of CD inflammation, laboratory markers, medication at the time of the US, and Montreal classification (MC) of disease, including age of onset, location of disease, and disease behaviour, were recorded for all patients and summarized in Table 1 [17].

### 2.2. Sonographic Evaluation

An US scan focused on the bowel was performed by experienced sonographers using either a Philips iU22 (Philips, Bothell, WA) or a Toshiba Aplio XG (Toshiba, Tokyo, Japan) ultrasound machine. Patients were instructed to fast overnight. A series of axial and longitudinal scans from the right flank to the left flank were performed to assess the bowel with addition of a high-frequency probe (7–9 MHz) for superior bowel wall resolution of regions of interest.

The US examinations were interpreted by one radiologist (SW) with more than 25 years of experience in bowel ultrasound. Standard features of CD inflammation were recorded, including mural thickening exceeding 3 mm, increased blood flow detected on CDI, increased mesenteric inflammatory fat, and lymphadenopathy. Stricture was considered to be present when thickened bowel wall with a rigid appearance and luminal compromise was identified concomitant with incomplete bowel obstruction, shown by increased proximal luminal calibre and fluid content with excess and dysfunctional peristalsis [18]. Other CD-related complications and incidental findings, unrelated to CD [9], were recorded.

Image storage included single-frame representative images and cineloop files to show relevant relationships and peristaltic activity.

### 2.3. Endoscopic Evaluation

Colonoscopies were performed by different endoscopists, using EC-3890Li adult and EC-3490LK paediatric endoscopes. Endoscopic reports and images were evaluated to determine the extent and activity of the disease. Reasons for incomplete examination or premature termination of the procedure were recorded. Disease activity was graded as normal (no evidence of disease activity), mild (evidence of aphthous ulceration ≤0.5 cm, absence of bridging inflammation, and no overt ulceration), or active, combining both moderate and severe changes, including ulceration >0.5 cm with oedema, erythema, and possibly bridging inflammation [19]. Disease activity at the anastomosis was recorded using Rutgeerts' score [20].

### 2.4. Change in Disease Management

In the ultrasound group, charts and source medical records were reviewed for any change in medical or surgical management occurring within two months of the US study, including any medication change, with initiation of a medication for treatment of IBD, initiation of antibiotics, or dose escalation of an existing IBD treatment; admission to hospital; and referral for therapeutic IBD surgical procedure (such as dilatation of stricture and bowel resection). In order to predominantly attribute a change in management to the US examination, we excluded patients with nonpenetrating active colonic disease detected at endoscopy or patients classified by Rutgeerts' score of i4 at the neoterminal ileum.

### 2.5. Statistical Analysis

Continuous numerical data are presented as mean (±standard deviation). A two-sample* t*-test or Mann-Whitney-Wilcoxon test is used for comparison of continuous variables between the endoscopy and ultrasound groups. Fisher's exact test is performed to detect differences in categorical variables between the two groups. The statistical software R (version 3.1.0, R Foundation for Statistical Computing, Austria) is used for all statistical analyses.

## 3. Results

### 3.1. Patient Demographics

**Figure 1:**
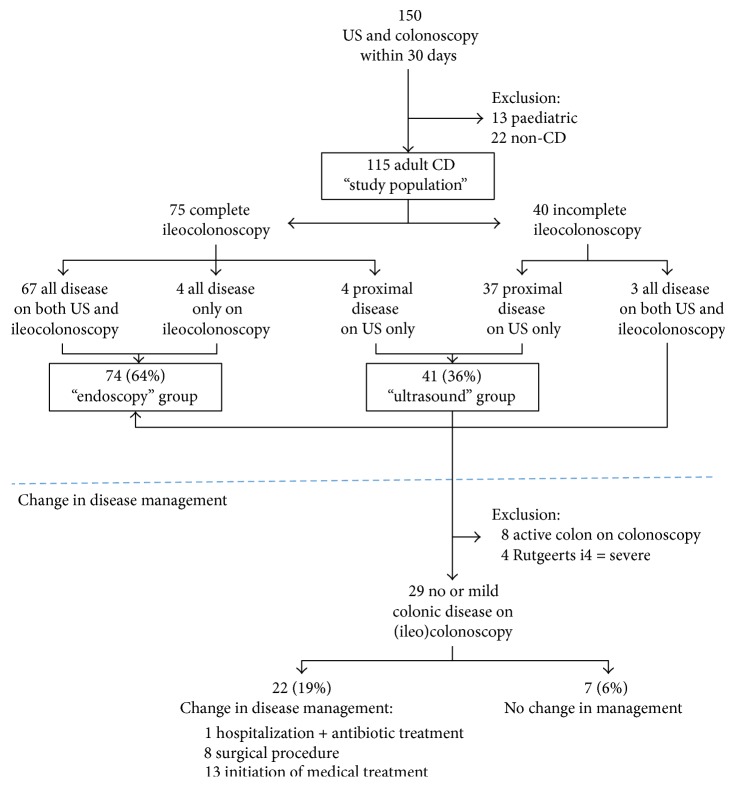
Schematic representation of the study population and change in disease management. 150 patients referred for intestinal ultrasonography (US) for suspected Crohn's disease (CD) and temporally related colonoscopy. Change in disease management is shown for the ultrasound group.

A total of 150 patients with suspected CD and temporally related US and colonoscopy examinations (within 30 days before or after), with an average time interval of nine days, were identified (Figure 1). Thirteen paediatric patients with CD and 22 adult patients without a confirmed diagnosis were excluded, resulting in a study population of 115 adult patients with CD. Table 1 includes the demographic information, location of disease, disease behaviour, and laboratory markers for the study population. Within the study population, complete ileocolonoscopy, with successful intubation of the TI, was achieved in 75 of 115 patients (65.2%).

#### 3.1.1. Endoscopy Group

Within the population with complete ileocolonoscopy, there are 67 patients with concordant US results (89%) and four (5%) with mild colonic disease shown only on colonoscopy (false negative US). These 71 patients comprise the major component of the endoscopy group (see Figure 1). However, three further patients with incomplete ileocolonoscopy, given premature termination of the procedure, are also included, since endoscopy identified the entire extent of disease with complete sonographic correlation (MC L2). Therefore, the total endoscopy group has 74 patients.

#### 3.1.2. Ultrasound Group

The 40 of 115 adult CD patients (34.8%) with incomplete ileocolonoscopy include 37 in whom a diseased segment of bowel was located either at the TI (MC L1/L3) or proximal to the reach of the endoscope (MC L4), fully detected on US only. These comprise the ultrasound group. Four additional patients with complete ileocolonoscopy exhibiting proximal small bowel disease were identified by US only (MC L4) and are also included, increasing the total ultrasound group to 41 patients.

**Figure 2:**
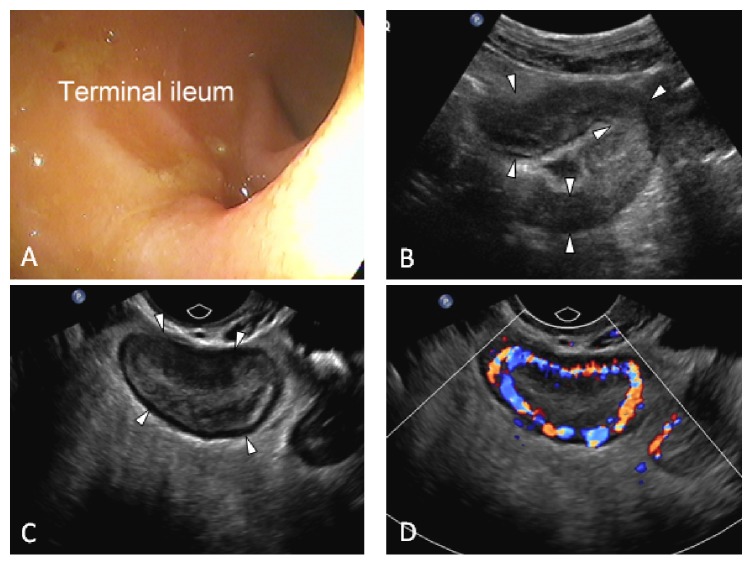
Negative ileocolonoscopy. 26-year-old female with 10-year history of Crohn's disease. Ileocolonoscopy shown in (A) is normal. (B) Transabdominal ultrasound shows a proximal angulated and thickened loop of ileum (arrowheads). (C) is an endovaginal ultrasound scan showing bowel wall thickening and abundant inflammatory fat with superior resolution. In (D) the addition of colour Doppler shows profuse mural vascularity. Interpretation: severe acute inflammation of a proximal ileal loop. Systemic steroids were initiated after ultrasound.

In the ultrasound group (41/115, 35.6%), narrowing of the intestinal lumen (MC B2) was the most common reason for preventing advancement of the endoscope (*n* = 32, 78.0%) including a stenotic ileocecal valve (ICV) in 15 cases (36.6%), a stenotic anastomosis in 8 cases (19.5%), terminal ileal stenosis in 5 cases (12.2%), and colonic stenosis in 4 cases (9.8%). Other causes of nonvisualisation of the diseased bowel segment included patient intolerance (*n* = 3, 7.3%) and need to prematurely terminate the procedure given disease severity (*n* = 2, 4.9%). Four additional patients (9.8%) had proximal small bowel disease beyond the reach of the standard ileocolonoscopy with skipping of the distal TI (MC L4) (Figure 2). US detected stenosis in 25 of the 32 cases (sensitivity: 78%; specificity: 100%).

### 3.2. Ultrasound Group Findings

**Figure 3:**
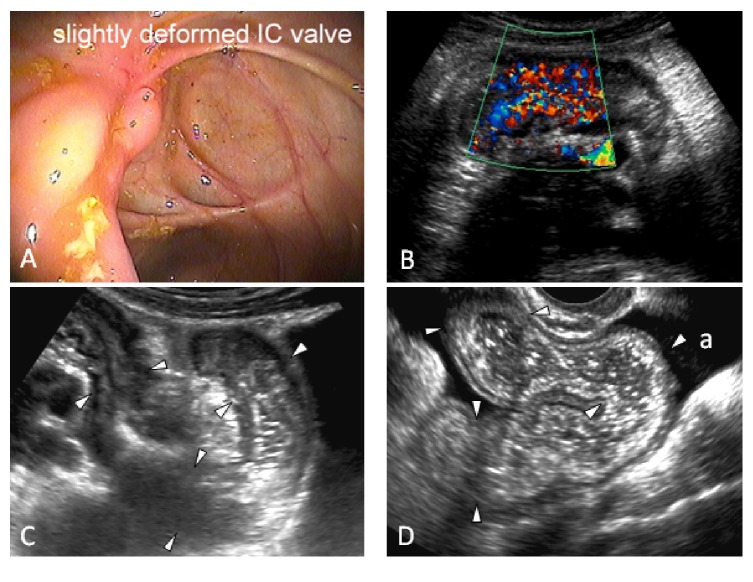
Stricture. 27-year-old female with no prior diagnosis of Crohn's disease with a failure to intubate the terminal ileum on colonoscopy due to a deformity at the ileocecal (IC) valve shown in (A). (B) An ultrasound image of the terminal ileum shows that it is thick-walled and hyperemic. (C) Additional ultrasound image shows a fixed acute angulation of the bowel. (D) shows the bowel proximal to the angulation with increased calibre and increased fluid content. There is ascites (a). Interpretation: hyper- and dysfunctional peristalsis on real-time imaging indicated acute inflammatory stricture with incomplete mechanical bowel obstruction. Biological therapy was initiated after ultrasound.

**Figure 4:**
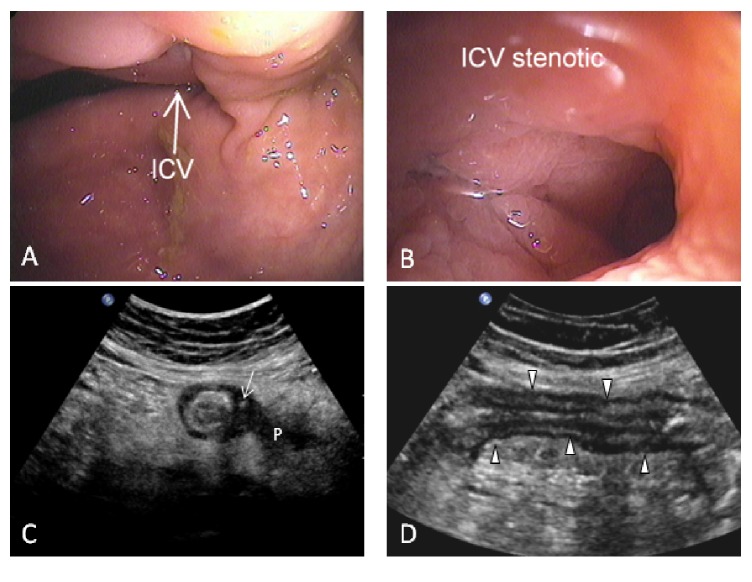
Complicated Crohn's disease on ultrasound only. 45-year-old female with a 2-year history of Crohn's disease. Colonoscopy failed to intubate the terminal ileum and did not detect any active disease. (A) shows the ileocecal valve (ICV) and (B) shows a close-up view of the orifice with limited view of the terminal ileum (TI). (C) An axial ultrasound image of the TI shows mural thickening and surrounding inflammatory fat. The wall is eccentrically thickened and shows intramural extraluminal air (arrow), suggestive of localised perforation. A poorly marginated hypoechoic zone within the inflammatory fat suggests a phlegmon (P). (D) is the corresponding sagittal image of the thickened TI. Interpretation: acute local perforation and phlegmon. After ultrasound, the patient was referred to surgery.

**Figure 5:**
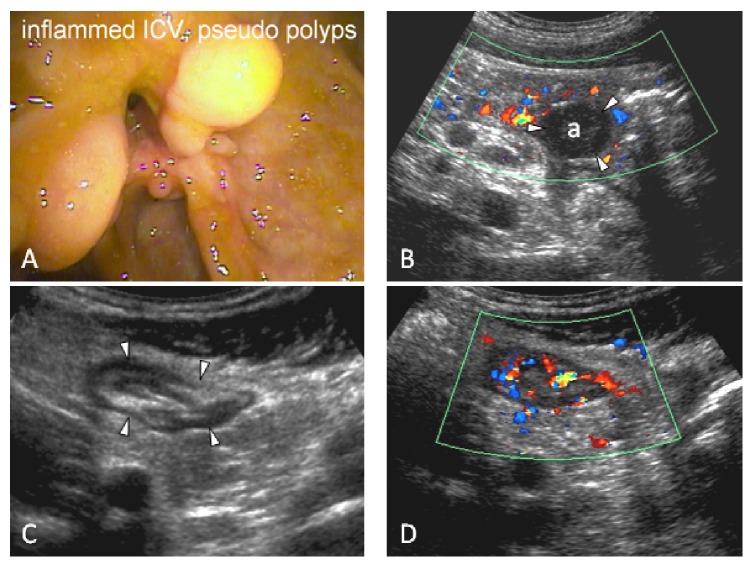
No prior diagnosis of Crohn's disease. 17-year-old male patient with no prior diagnosis of Crohn's disease. Colonoscopy is incomplete with a failure to intubate the inflamed ileocecal valve, shown in (A), with pseudopolyps. (B) Transabdominal ultrasound shows an interloop abscess cavity (a). (C) shows a thickened terminal ileum in axial view. The length of involvement (not shown) is 20 cm. (D) shows hyperaemia on colour Doppler. Interpretation: acute inflammation of the terminal ileum with a small interloop abscess. Four days after the ultrasound, the patient was admitted to hospital for intravenous antibiotic therapy.

Pathology shown on US but not appreciated on ileocolonoscopy included the following: abnormal bowel with segmental wall thickening above 3 mm in all patients (*n* = 41), increased hyperaemia on CDI (*n* = 36), presence of inflammatory fat (*n* = 37), and lymphadenopathy (*n* = 24). Further, US showed CD-related complications in 26 patients, including the 25 strictures (Figure 3), 7 phlegmons (Figure 4), 6 fistulas, 5 local perforations (Figure 4), and 3 abscesses (Figure 5). Three of the 26 patients showing complications at ultrasound had their diagnosis within a year. An additional 5 patients (12%) in the ultrasound group had no diagnosis of CD prior to their US examination, showing complicated disease at presentation, necessitating surgery with confirmation of diagnoses in all. The remaining 18/26 (69%) patients had a mean disease duration of 15.3 years. Previously undetected incidental findings unrelated to CD were also found in nine patients, including one renal cell carcinoma.

Of the 41 patients in the ultrasound group, 8 (20%) had moderately or severely active nonpenetrating colonic disease visualised on endoscopic assessment, and a further 4 (10%) patients, with endoscopic failure to intubate a surgical anastomosis, were graded as Rutgeerts' score of i4 (narrowed and inflamed anastomosis). Combined, these 12 patients were not investigated for changes in disease management, as these could not be confidently credited to US alone as active disease was also detected endoscopically. Nonetheless, based on their colonoscopy exam, the extent and severity of the undetected proximal bowel segment remains unknown in this group. Following necessary exclusions, the remaining 29/41 (71%) patients were assessed for changes in management which were noted in 22/29 (76%) at an average of 21.7 days (range: 2–60) following the US examination. Thirteen patients, 13/29 (45%), underwent initiation of medical therapy including topical steroids (*n* = 2), systemic steroids (*n* = 1), mesalamine (*n* = 1), combination of steroid and antibiotic (*n* = 1) or antibiotic alone (*n* = 2), combination of steroid and immunomodulator (*n* = 2), and biologic therapy alone (*n* = 2) or in combination with an immunomodulator (*n* = 1), and one patient underwent a dose escalation of biologic medication.

Surgical referral or management within two months was undertaken for eight patients, 8/29 (28%), including 6 patients with penetrating disease diagnosed on US. Finally, one patient was admitted to hospital for antibiotic treatment. Further, 7/29 patients (24%) underwent no change in therapy.

## 4. Discussion

In this study, we evaluate patients with temporally related US and ileocolonoscopy. We identified 41/115 (36%) where abnormal bowel is assessed on ultrasound alone, not shown on ileocolonoscopy. In our study, 22 of 115 (19%) had a management change potentially owing to US findings. Further, there were 5 patients in the ultrasound group with no prior diagnosis of CD before the ultrasound examination, showing abnormal and thickened proximal small bowel. All five patients had their final diagnosis confirmed by surgery/pathology of the specimen. Undiscovered complications and a single incidental carcinoma of the kidney were additionally shown in the ultrasound group.

The ileum is the most commonly affected anatomic site in CD, and disease location tends to remain stable over time [21]. Even in a healthy population, there are limits to endoscopic evaluation of the ileum, with nonvisualisation of the ileum occurring in clinical practice in up to one in five patients [6]. Nevertheless, ileocolonoscopy is considered the gold standard in Crohn's disease; however, the limits to endoscopic evaluation are also well established, with up to 30% of those with a normal ICV exhibiting an abnormal proximal TI [22]. Upon intubation of the TI, a normal endoscopic appearance of the mucosa may be falsely assuring [6]. Patients with more severe IBD are at higher risk of incomplete colonoscopy as well as complications such as perforation from the procedure [23, 24]. In one-third of all patients included in this study, US examination revealed more severe disease than shown on endoscopy, thus facilitating the introduction of potentially earlier or more aggressive management than would otherwise be suggested based solely on endoscopic findings. Accurate mapping of the extent and severity of disease on imaging allows for the best treatment choice, potentially altering the patients' long-term outcome [25].

CD evolves over time with increasing risk of transmural damage. Cross-sectional imaging is essential at the time of diagnosis, but given variable response to therapy and tendency towards loss of response, US should also be undertaken at regular intervals during follow-up [7]. Twenty-six of the 41 patients, 63% of the ultrasound group, exhibited complications including fistulae, stenoses, and/or perforations. 69% of these patients had a mean disease duration of 15.3 years and were evaluated for routine follow-up, illustrating the importance of interval evaluation.

Incomplete ileocolonoscopy occurred in 40/115 (35%) of study patients. This is higher than existing published data; however, significant population differences and bias likely exist [6, 26]. The patients included from this center reflect a tertiary referral population with a high disease burden and severity and presence of complications including stenoses, as opposed to overall limitations due to technical difficulty. Further, our entire study population is comprised of patients with active disease as opposed to comparable studies [6, 26]. Lastly, we chose to limit the interval between endoscopy and ultrasound to 30 days to minimize influence of fluctuating disease activity. However, a short interval between procedures generally occurs in urgent cases and cases after incomplete endoscopy. Our population, therefore, reflects a bias based on a higher likelihood of urgent physician requests for imaging.

Rates of isolated proximal small bowel disease (MC L4) are variable, between 1 and 19% in cohort- or population-based studies [27, 28]. In our study, 4/75 (5%) patients with complete ileocolonoscopy showing a normal TI had proximal disease discovered on US. This is comparable to Jensen et al. [26] showing additional inflammation on CTE/MRE or capsule endoscopy in 3/43 (7%) patients with normal colon and successful visualisation of the normal TI. Samuel et al. [6] found active intestinal disease in 13/67 (19%) with known small bowel CD and normal-appearing TI on endoscopy. However, this population was highly selected and therefore not comparable.

There is no existing gold standard for inflammation of the small bowel [29] and surgery is considered gold standard for detection of fistulas [7]; however, cross-sectional imaging provides essential additional information to colonoscopy, particularly of the small bowel, which is mostly inaccessible by conventional endoscopic means. Although CT enterography exhibits excellent resolution and good diagnostic accuracy, there is increasing awareness of potential risks imparted by ionizing radiation. Thus, given safety concerns, repeat use of CT should be limited, particularly in this commonly young population. MR is safe and accurate; however, it is expensive and its access limited in many centers. Ultrasound has demonstrated equivalent accuracy to MR for inflammation and fistula detection [30, 31] and should thus be considered as a safe diagnostic alternative, depending on local expertise. Despite the established equivalency of intestinal US compared to MR and CT [7, 8], US of the bowel is not widely available globally.

In the ultrasound group, US identified 25 strictures defined by luminal compromise and proximal dilatation [18]. Endoscopy, by some considered gold standard [32], demonstrated an additional 7 stenoses on the basis of narrowing of the lumen. Lack of complete agreement is not surprising by reason of unequal definitions [18, 33] but is similar to prior studies [32]. US has the distinct advantage of real-time functional assessment of the bowel and strictures, showing proximal dilatation and dysfunctional peristalsis. Detection of stenoses on US may be further increased with the addition of oral contrast [34–36]. Intestinal dilatation is influenced by the amount and administration method of oral contrast used but not the detection rate of stenoses [35].

Attributing change in management to a specific entity or test (in this case US) is challenging given the complexity and variety of phenotypes of this chronic disease. We recognise that treatment choice and clinical management are complex and involve weighing multiple factors, including clinical symptoms, disease duration and character, consideration of disease behaviour and prior surgical interventions, blood work with biochemical markers such as C-reactive protein and fecal calprotectin, endoscopy with associated histology, and cross-sectional imaging. Retrospective review, however, did not provide consistent temporally related biochemical and clinical disease activity assessment and thus could not be reliably included in this analysis. The reported influence of cross-sectional imaging on the management of IBD is significant, ranging from 51 to 61% [37–39]; however, little has been reported regarding the influence of US compared to other modalities. The average likely impact of US was realized in this study within 21 days, with 22/115 patients (19%) having US as the predominant factor for change in management. However, US may also have contributed to clinical management in all but the 4 false negative cases, but to a lesser extent, since colonoscopy showed identical findings or severe colonic disease. As a limitation, some patients also had other imaging examinations and symptoms confirming our US findings not included in this review. A recent prospective study by Bruining et al. [38], on change in disease management after CT enterography, found that 31/145 patients (21%) had an additional or increase in medication, surgical referral, or a new established involvement of proximal disease or presence of an abscess. Although our findings are from a selected retrospective population with the potential for more severe disease, they reflect the real-world limitations of endoscopy and thus the significant potential contribution of US as an essential adjunct.

In conclusion, ultrasound may provide an important addition to endoscopy for mapping disease extent and severity in CD patients not only at diagnosis but also in the follow-up evaluation of disease activity regardless of clinical symptoms. We demonstrate that US likely makes a significant contribution to correct classification of disease extent and severity of patients with active CD undergoing ileocolonoscopy, particularly when the endoscopic examination is not complete. US has a potential impact on disease management in a fifth of those patients, revealing complications such as strictures and penetrating disease. We therefore submit US is an excellent cross-sectional imaging option in the diagnosis and monitoring of patients with Crohn's disease, as it is an inexpensive, accurate, and patient-friendly modality, which should rival both CT and MR. Further prospective studies are needed to establish the true impact of ultrasound on disease management.

## Figures and Tables

**Table 1:** Demographics, Montreal classification, and laboratory markers of ultrasound and endoscopy groups.

	Ultrasound group	Endoscopy group	*p* value
Demographic information	*n* = 41	*n* = 74	
Age (y)	40.2 ± 13.8	37.5 ± 13.7	.31
Sex (female)	27 (66)	43 (58)	.43
Previous surgery	14 (34)	21 (28)	.53
New disease	13 (32)	31 (41)	.32
Duration of disease (y)	10.2 ± 11.9	7.4 ± 9.5	.23
B1 (y)	3.3 ± 5.5	6.7 ± 9.1	
B2/B3 (y)	11.5 ± 11.4	8.9 ± 10.4	
Age at diagnosis			.31
A1	5 (12)	7 (10)	
A2	26 (63)	48 (65)	
A3	10 (24)	19 (26)	
Location of disease			.67
L1	23 (56)	24 (32)	
L2	5 (12)	30 (41)	
L3	12 (29)	20 (27)	
L4	1 (2)	0 (0)	
Disease behaviour			.52
B1	7 (17)	52 (70)	
With perianal disease	0	10	
B2	20 (49)	8 (11)	
With perianal disease	1	1	
B3	14 (34)	14 (19)	
With perianal disease	0	2	
Laboratory markers			
C-reactive protein (mg/L)	27.92 ± 40.4	37.6 ± 59.9	.36
Albumin (g/L)	32.85 ± 5.7	30.7 ± 7.2	.12
Hemoglobin (g/L)	123.57 ± 20.0	119.95 ± 21.4	.39

Continuous values are mean ± SD; discrete values are *n* (%). *Montreal classification* [17]. *Age of onset*: A1: <17 y; A2: 17–40 y; A3: above 40 y. *Location*: L1: distal ileum; L2: colonic; L3: ileocolonic; L4: upper disease. *Disease behaviour*: B1: nonstricturing and nonpenetrating; B2: stricturing; B3: penetrating.
